# Balancing confidentiality and care coordination: challenges in patient privacy

**DOI:** 10.1186/s12912-024-02231-1

**Published:** 2024-08-15

**Authors:** Ateya Megahed Ibrahim, Hassanat Ramadan Abdel-Aziz, Heba Ali Hamed Mohamed, Donia Elsaid Fathi Zaghamir, Nadia Mohamed Ibrahim Wahba, Ghada. A. Hassan, Mostafa Shaban, Mohammad EL-Nablaway, Ohoud Naif Aldughmi, Taghreed Hussien Aboelola

**Affiliations:** 1 College of Nursing, Prince Sattam Bin Abdulaziz University, Alkarj, Saudi Arabia; 2 Family and Community Health Nursing Department, Faculty of Nursing, Port Said University, Port Said City, Port Said 42526 Egypt; 3 Gerontological Nursing Department, Faculty of Nursing, Zagazig University, Zagazig, Egypt; 4 Community Health Nursing Department, Faculty of Nursing, Mansoura University, Mansoura City, Dakahlia Egypt; 5 Pediatric Nursing Department, Faculty of Nursing, Port Said University, Port Said City, 42526 Egypt; 6 Psychiatric Nursing and Mental Health Department, Faculty of Nursing, Port Said University, Port Said, 42526 Egypt; 7 Pediatric Nursing Department, Faculty of Nursing, Menoufia University, Shibin el Kom, Egypt; 8 Community Health Nursing Department, College of Nursing, Jouf University, Sakaka, Al Jouf 72388 Saudi Arabia; 9 Department of Basic Medical Sciences, College of Medicine, AlMaarefa University, P.O.Box 71666, 11597 Riyadh, Saudi Arabia; 10 Department of Medical and Surgical Nursing, Northern Border University, Arar, Saudi Arabia; 11 Nursing Leadership Department, Nursing College, Northern Border University, Arar, Saudi Arabia

**Keywords:** Care coordination, Digital health, HIPAA compliance, Nurses, Patient confidentiality, Privacy frameworks

## Abstract

**Background:**

In the digital age, maintaining patient confidentiality while ensuring effective care coordination poses significant challenges for healthcare providers, particularly nurses.

**Aim:**

To investigate the challenges and strategies associated with balancing patient confidentiality and effective care coordination in the digital age.

**Methods:**

A cross-sectional study was conducted in a general hospital in Egypt to collect data from 150 nurses across various departments with at least six months of experience in patient care. Data were collected using six tools: Demographic Form, HIPAA Compliance Checklist, Privacy Impact Assessment (PIA) Tool, Data Sharing Agreement (DSA) Framework, EHR Privacy and Security Assessment Tool, and NIST Cybersecurity Framework. Validity and Reliability were ensured through pilot testing and factor analysis.

**Results:**

Participants were primarily aged 31–40 years (45%), with 75% female and 60% staff nurses. High compliance was observed in the HIPAA Compliance Checklist, especially in Administrative Safeguards (3.8 ± 0.5), indicating strong management and training processes, with an overall score of 85 ± 10. The PIA Tool showed robust privacy management, with Project Descriptions scoring 4.5 ± 0.3 and a total score of 30 ± 3. The DSA Framework had a mean total score of 20 ± 2, with Data Protection Measures scoring highest at 4.0 ± 0.4. The EHR assessments revealed high scores in Access Controls (4.4 ± 0.3) and Data Integrity Measures (4.3 ± 0.3), with an overall score of 22 ± 1.5. The NIST Cybersecurity Framework had a total score of 18 ± 2, with the highest scores in Protect (3.8) and lower in Detect (3.6). Strong positive correlations were found between HIPAA Compliance and EHR Privacy (*r* = 0.70, *p* < 0.05) and NIST Cybersecurity (*r* = 0.55, *p* < 0.05), reflecting effective data protection practices.

**Conclusion:**

The study suggests that continuous improvement in privacy practices among healthcare providers, through ongoing training and comprehensive privacy frameworks, is vital for enhancing patient confidentiality and supporting effective care coordination.

## Background

Digital technology has significantly transformed healthcare, enhancing care coordination and improving patient outcomes. However, this transformation brings forth critical challenges, particularly in balancing the imperatives of confidentiality and efficient care coordination [1]. The intersection of these essential elements, patient privacy and the seamless sharing of information among healthcare providers requires a nuanced approach to ensure ethical and legal compliance while optimising patient care [2].

Confidentiality in healthcare is foundational, rooted in bioethics principles and protected by laws such as the Health Insurance Portability and Accountability Act (HIPAA) in the United States [3]. HIPAA establishes national standards to safeguard individuals' medical records and other personal health information, emphasising the importance of privacy in the digital age [4]. As digital technologies become more embedded in healthcare practices, ensuring compliance with these standards while facilitating the necessary flow of information for care coordination becomes increasingly complex [5]. Care coordination, defined as the deliberate organisation of patient care activities to facilitate the appropriate delivery of health services, is essential for achieving high-quality healthcare [6]. Effective care coordination requires timely and accurate sharing of patient information among various healthcare providers, which can be challenging when strict confidentiality rules are in place [7, 8].

Nurses are responsible for ensuring patient information is shared accurately and promptly with other healthcare team members to facilitate effective care coordination [9]. However, they must also strictly adhere to confidentiality protocols to protect patient privacy. This dual responsibility can create significant tension and complexity in their daily practice. Nurses must navigate varying levels of digital literacy, differing institutional policies on information sharing, and the ever-present risk of data breaches or inadvertent disclosures [10]. Furthermore, the pressure to use electronic health records (EHRs) efficiently while maintaining patient trust and confidentiality adds to the complexity of their role [11]. These challenges highlight the need for robust training, clear guidelines, and support systems to help nurses effectively manage the delicate balance between confidentiality and care coordination.

## Introduction

Electronic Health Records (EHRs) are central to enhancing care coordination by providing comprehensive, real-time access to patient health information, facilitating more informed decision-making and continuity of care [12]. However, digitising health records also raises significant privacy concerns, increasing the risk of unauthorised access and data breaches [13]. Thus, healthcare providers must implement robust security measures to protect patient data while ensuring it is accessible to authorised personnel when needed [14].

Although telehealth offers significant benefits in terms of accessibility and convenience, particularly for patients in remote or underserved areas, it further complicates the balance between confidentiality and care coordination [15, 16]. It introduces challenges in maintaining patient privacy, preventing breaches, and safeguarding patient data [17]. Additionally, there is a critical issue concerning who has access to this information, which raises justice concerns about equitable access and safeguarding patient data. Addressing these concerns involves implementing robust access controls and consistently applying privacy measures across all telehealth platforms [18]. Patient consent is another critical factor for maintaining patient trust and ensuring that individuals know how their information will be used and shared [19]. However, the complexity of digital health systems can make it difficult for patients to fully understand the implications of consent, particularly regarding sharing their data across multiple platforms and providers [20–22].

Nurses play a pivotal role in balancing confidentiality and care coordination in the digital age, acting as guardians of patient privacy and key facilitators of information sharing. Their unique position on the front lines of patient care requires them to navigate complex ethical and practical challenges. Nurses are often responsible for inputting and accessing data within EHRs, making their adherence to privacy protocols crucial for protecting patient information [23]. Additionally, they serve as critical links in the care coordination chain, ensuring that relevant health information is accurately communicated among various healthcare providers to support comprehensive patient care [24]. As the healthcare landscape becomes increasingly digital, ongoing education and training for nurses in the technological aspects of EHRs and the ethical implications of data handling are essential [25].

Healthcare institutions must adopt comprehensive policies and technological solutions to manage the dual imperatives of confidentiality and care coordination [26] to help mitigate the risks associated with data breaches and unauthorised access [27]. Interoperability between different healthcare systems is another significant challenge, and efforts to develop and implement interoperable systems are essential for balancing the need for information sharing with protecting patient privacy [28, 29] ensuring that patient welfare remains the primary focus [30]. In addition, empowering patients to take an active role is crucial, and education and communication strategies are essential for helping patients understand their rights and measures to protect their privacy [31].

Healthcare institutions must adopt comprehensive policies and frameworks to manage the dual imperatives of confidentiality and care coordination. These policies should include guidelines for data security, patient consent, and the ethical use of health information [26]. Technological solutions such as encryption, anonymisation, and secure access controls are crucial for protecting patient data in digital systems. These technologies help mitigate the risks associated with data breaches and unauthorised access, ensuring that sensitive information remains secure while being accessible to those who need it for patient care [27].

Interoperability between different healthcare systems is another significant challenge. The lack of standardised protocols for data exchange can hinder effective care coordination and increase the risk of privacy breaches [28]. Efforts to develop and implement interoperable systems are essential for balancing the need for information sharing with the protection of patient privacy [29]. Ethical frameworks must account for the potential benefits and harms of information sharing, ensuring that patient welfare remains the primary focus [30].

Patient engagement is also crucial in this context. Empowering patients to take an active role in their healthcare, including decisions about their information, can enhance trust and improve outcomes. Education and communication strategies are essential for helping patients understand their rights and the measures in place to protect their privacy [31].

In conclusion, balancing confidentiality and care coordination in the digital age is a complex but essential task for modern healthcare. Ensuring patient privacy while facilitating the necessary flow of information for care coordination requires a multifaceted approach that includes robust technological solutions, comprehensive policies, ongoing education and training, and active patient engagement. By addressing these challenges, healthcare providers can improve patient outcomes and maintain public trust in the healthcare system.

### Significance of the study

This study is significant as it addresses the critical intersection of confidentiality and care coordination in the rapidly evolving digital healthcare landscape. By examining the practices and perceptions of healthcare professionals, particularly nurses, the research sheds light on how effectively these individuals face challenges posed by digital technologies while ensuring patient privacy. Understanding the dynamics of confidentiality and care coordination informs best practices and enhances the development of training programs and institutional policies to improve patient outcomes.

The findings of this study have several practical applications. Institutions can design targeted training programs focusing on both technical skills and ethical considerations to educate nurses on safeguarding patient information while ensuring efficient care coordination. Insights can inform the creation or revision of data security and patient consent guidelines, ensuring staff understand the importance of maintaining patient privacy and secure data sharing. Additionally, the study promotes integrating advanced security features in Electronic Health Record (EHR) systems, balancing data protection with necessary access for care coordination. This research can build patient trust by highlighting best practices and effective strategies for balancing confidentiality and care coordination, leading to better cooperation and health outcomes. Furthermore, these findings can support the development of standardised protocols for telehealth services, ensuring consistent privacy measures across platforms and improving equitable access to care.

### Aim of the study:

To investigate the challenges and strategies associated with balancing patient confidentiality and effective care coordination in the digital age.

### Research questions:

1. What are healthcare providers' primary challenges in maintaining patient confidentiality while utilising digital health technologies for care coordination?
2. How do different privacy assessment tools and frameworks impact the balance between patient confidentiality and the efficiency of care coordination in digital healthcare environments?
3. What best practices can be implemented to maintain patient privacy without compromising care coordination in the digital age?

### Theoretical framework

The theoretical framework for this study incorporates several key theories to understand the balance between confidentiality and care coordination in the context of digital health technologies.

Privacy Regulation Theory, proposed by Westin32, emphasises that privacy is a fundamental human right involving control over the extent, timing, and circumstances of sharing oneself with others. This theory is crucial for understanding the importance of maintaining patient confidentiality in healthcare settings. It underscores the need for stringent privacy measures to build and maintain trust between patients and healthcare providers. Using this theory, the study addresses the first research question concerning healthcare providers' challenges in maintaining patient confidentiality. It offers a conceptual foundation for exploring the importance of privacy in patient-provider relationships and the implications of privacy breaches in digital health environments.

Health Information Technology (HIT) Adoption Framework, as described by Venkatesh et al.33, examines factors influencing the adoption of health information systems, such as perceived usefulness, ease of use, and institutional support. This framework is relevant for understanding how healthcare professionals, particularly nurses, adopt and utilise digital technologies while managing patient privacy. It addresses the second research question about how privacy assessment tools and frameworks impact the balance between patient confidentiality and care coordination. The framework provides insights into the factors that facilitate or hinder the adoption of digital health technologies, which is essential for effective care coordination.

The Technology Acceptance Model (TAM), proposed by Davis 34, explains how users accept and use technology, emphasising perceived ease of use as primary determinants. TAM is pertinent for understanding healthcare professionals' attitudes toward digital health technologies and how these attitudes influence their adoption and usage. This model supports the exploration of the third research question regarding best practices for ensuring patient privacy without compromising care coordination. It provides a basis for developing strategies to enhance the acceptance and effective use of digital health technologies among healthcare providers.

Ethical Decision-Making Framework, based on Beauchamp and Childress's principles of biomedical ethics—autonomy, beneficence, non-maleficence, and justice—guides ethical considerations in healthcare [35]. This framework is integral for evaluating the ethical implications of maintaining confidentiality while promoting care coordination. It helps address the ethical challenges identified in the first research question. It supports the development of best practices outlined in the third research question. This framework ensures that ethical principles guide decisions about information sharing and patient privacy in digital health environments. Additionally, Grady's Ethical Framework for Health Informatics emphasises integrating ethical considerations into the design and use of health information technologies, ensuring that privacy and care coordination are complementary goals [36].

Diffusion of Innovations Theory, proposed by Rogers [37], explains how new ideas and technologies spread within a social system, focusing on communication channels, social systems, and the attributes of innovations. This theory is relevant for understanding how digital health innovations are adopted in healthcare settings and their impact on confidentiality and care coordination. It helps explore the challenges of adopting digital health technologies addressed in the first research question. It supports identifying best practices for integrating new technologies into healthcare practice, as addressed in the third research question. The theory provides insights into the adoption process and the factors influencing the successful integration of innovations into healthcare practice.

## Conceptual framework

The conceptual framework for this study explores the dynamic interaction between confidentiality, care coordination, and the utilisation of digital health technologies, with insights drawn from several theoretical perspectives. Confidentiality protects patient information from unauthorised access, which is critical for maintaining trust in healthcare settings [38]. Privacy Regulation Theory emphasises that privacy is a fundamental human right, focusing on controlling the extent, timing, and circumstances of sharing personal information. This theory underlines the necessity of robust privacy measures to ensure patient data security and build trust between patients and healthcare providers [32].

Care coordination refers to effectively managing and integrating patient care across different healthcare providers and settings. It involves ensuring that care is seamless and that information is shared appropriately among various stakeholders to provide comprehensive and continuous care [39]. The Health Information Technology (HIT) Adoption Framework sheds light on how factors such as perceived usefulness, ease of use, and institutional support influence the adoption of health information systems. This framework helps us understand how healthcare professionals integrate digital technologies into their workflows while managing patient privacy and enhancing care coordination [33].

Using digital health technologies includes tools such as electronic health records (EHRs) and telehealth platforms that facilitate communication, information sharing, and care coordination. These technologies are critical for modern healthcare delivery but also raise challenges related to confidentiality [1, 2]. The Technology Acceptance Model (TAM) provides a lens through which to examine how perceived ease of use and perceived usefulness affect the acceptance and effective use of these technologies. Understanding healthcare professionals' attitudes towards these tools is crucial for improving their integration and addressing potential barriers to technology adoption, which impacts confidentiality and care coordination [34].

The Ethical Decision-Making Framework, guided by Beauchamp and Childress's principles—autonomy, beneficence, non-maleficence, and justice—offers a foundation for evaluating the ethical implications of maintaining confidentiality while promoting care coordination. This framework helps ensure that information-sharing decisions respect patient autonomy and adhere to ethical standards, balancing privacy with the need for effective care [35, 36].

The Code of Ethics for Nurses further reinforces the importance of privacy by setting ethical guidelines for protecting patient information. This code ensures that nurses' practices align with ethical and legal standards, providing a practical framework for maintaining confidentiality while coordinating care effectively [14, 17].

Lastly, Diffusion of Innovations Theory explains how new technologies spread within healthcare systems, emphasising the roles of communication channels, social systems, and the attributes of innovations. This theory helps us understand how digital health innovations are adopted and how they impact the balance between confidentiality and care coordination. It provides insights into the factors influencing the successful integration of new technologies [37].

**Fig. 1:**
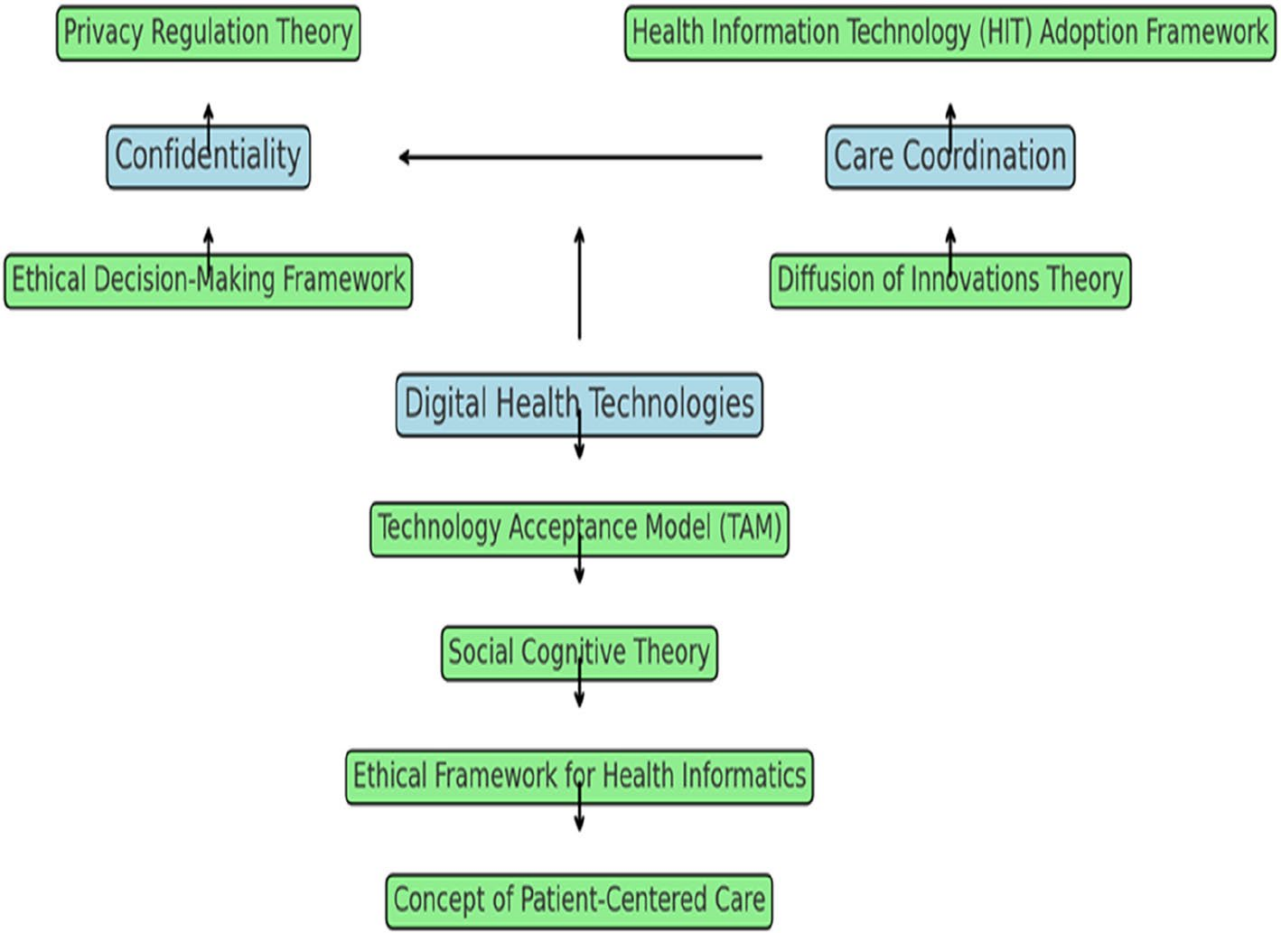
Balancing confidentiality and care coordination in digital health

The conceptual framework integrates these theories to comprehensively understand how confidentiality, care coordination, and digital health technologies interact. Each theory provides unique insights into the challenges and solutions of maintaining patient privacy while improving care coordination in a digital healthcare environment (Fig. 1).

## Methods

This cross-sectional study was conducted at General Hospital in Egypt to evaluate the balance between patient confidentiality and care coordination in the digital age. Data was collected from a sample of nurses working in various departments in the hospital. The recruitment process entailed inviting all eligible nurses through internal communication channels, such as email and notice boards, with detailed information about the study and the importance of their participation. A total of 150 nurses were needed to achieve a representative sample, calculated using the formula for sample size estimation for a finite population: *n* = z2 * p * (1—p)/e2 [40], where Z was the Z-value (1.96 for a 95% confidence level), p was the expected prevalence (assumed at 0.5 for maximum sample size), and e was the margin of error (0.05). The inclusion criteria for the study included nurses working at Damietta General Hospital for at least six months, directly involved in patient care, and consented to participate. Nurses on extended leave, such as maternity or sick leave, or those involved in administrative roles without direct patient care responsibilities were excluded from the study.

### Data collection tools

Six tools were used for data collection.

### Demographic form

The demographic questionnaire for this study was designed following a comprehensive review of relevant literature and studies and included variables such as age, gender, marital status, current job title/position at General Hospital, years of nursing experience, highest nursing qualification attained, training received on patient confidentiality and digital health technologies, and primary language of communication in the workplace. These variables were chosen to provide a comprehensive profile of the participating nurses, enabling a deeper analysis of their perceptions and practices concerning patient confidentiality and care coordination in the digital healthcare environment.

### Health insurance portability and cccountability act (HIPAA) compliance checklist

The Health Insurance Portability and Accountability Act (HIPAA) is a regulatory framework enforced by the U.S. Department of Health and Human Services (HHS) to safeguard patient privacy and secure health information [41]. Healthcare providers use the HIPAA Compliance Checklist to ensure adherence to regulations, protecting patient information from unauthorised access and breaches. The HIPAA Compliance Checklist was chosen for its comprehensive approach to ensuring regulatory compliance and its ability to provide quantifiable data on privacy practices to identify best practices for maintaining patient confidentiality and enhancing care coordination by evaluating how well healthcare facilities implement privacy measures in the context of digital technologies. Components include administrative safeguards, such as security management processes and workforce training; physical safeguards, like facility access controls and workstation security, and technical safeguards including access control and audit controls. The checklist also covers organisational requirements such as business associate contracts and documentation of policies and procedures. The checklist evaluates adherence using a scoring system that rates each component from 0 (non-compliant) to 4 (fully compliant), with a total score ranging from 0 to 100.

### Privacy impact assessment (PIA) tool

The Privacy Impact Assessment (PIA) tool, as detailed by Wright and De Hert [42], is used to identify and mitigate risks and ensure compliance with data protection regulations by thoroughly evaluating how information is collected, used, shared, and stored. The PIA tool typically includes sections on project descriptions, methods of data collection, practices for data usage and sharing practices, data storage and security strategies, identification of potential privacy risks, and methods for mitigating these risks. Each section is scored on a scale from 0 (non-compliant) to 5 (fully compliant), resulting in a total score range from 0 to 35. The PIA tool is chosen to identify and manage privacy risks, which aligns with the study's aim to balance confidentiality and care coordination. It helps evaluate how effectively privacy measures are integrated into new systems, thus ensuring that privacy concerns are proactively addressed and managed.

### Data sharing agreement (DSA) framework

The Data Sharing Agreement (DSA) framework [43] establishes clear protocols to ensure data privacy and security while facilitating effective care coordination to enhance patient care and comply with legal requirements. Key components of the DSA framework include defining the purpose of data sharing, specifying the types of data to be shared, outlining the roles and responsibilities of involved parties, implementing robust data protection measures, managing consent appropriately, and establishing terms for data use and retention. Regarding compliance evaluation, each section of the DSA framework was assessed on a scale from 0 (indicating non-compliance) to 4 (indicating full compliance), resulting in a total score range from 0 to 24. The DSA framework is chosen for its structured approach to managing data sharing while ensuring privacy and security. It supports the study's aim of balancing confidentiality with effective care coordination by providing a comprehensive system for managing data-sharing agreements.

### Electronic health record (EHR) privacy and security assessment tool

The Electronic Health Record (EHR) Privacy and Security Assessment Tool [44] is critical for evaluating EHR systems' privacy and security features. This tool ensures that EHR systems adhere to regulations and best practices, protecting patient information against unauthorised access and breaches. Key components evaluated by the assessment tool include access controls, encryption methods, audit trail functionalities, measures for maintaining data integrity, and protocols for incident response. In terms of scoring, each component was typically rated on a scale from 0 (indicating non-compliance) to 5 (indicating full compliance), resulting in a total score range from 0 to 25. This assessment tool is chosen for its comprehensive approach to evaluating EHR systems' security and privacy features, aligning with the study's aim of ensuring effective privacy protection while facilitating care coordination.

### National institute of standards and technology (NIST) cybersecurity framework

The National Institute of Standards and Technology (NIST) Cybersecurity Framework [45] is a foundational tool healthcare organisations, including nursing staff, use to enhance and assess their cybersecurity measures. Key components of the NIST Cybersecurity Framework include five core functions: Identify, Protect, Detect, Respond, and Recover. Each function incorporates specific categories and subcategories detailing activities and best practices for cybersecurity. Each core function can be assessed on a scale from 0 (indicating not implemented) to 4 (indicating fully implemented), resulting in a total score range from 0 to 20. This framework is selected for managing cybersecurity risks, aligning with the study's aim of safeguarding patient information while ensuring effective care coordination.

### Validation and reliability

In the preliminary phase of this study, a pilot test was conducted involving 10% of the total nurses, equivalent to 10 individuals, using the newly introduced data sharing agreement (DSA) framework, the electronic health record (EHR) privacy and security assessment tool, and the National Institute of Standards and Technology (NIST) Cybersecurity Framework. These participants were excluded from the final sample size to mitigate any potential bias from their prior exposure to the research instruments, ensuring the integrity of the results. During the pilot phase, a crucial step involved implementing factor analysis. This statistical technique was employed to rigorously examine the relevance and accuracy of each component within the research instruments. Following the pilot study, the insights gained from factor analysis informed the decision-making process for the final study. The same factor analysis methodology was applied to the remaining nurses who were not part of the pilot study.

Additionally, content validity was rigorously employed as a methodological approach to validate the measurement instruments used in this study. Specifically, for the data sharing agreement (DSA) framework, electronic health record (EHR) privacy and security assessment tool, and National Institute of Standards and Technology (NIST) Cybersecurity Framework, content validity procedures were implemented to ensure that the items within these instruments accurately and comprehensively captured the intended constructs. Experts possessing considerable knowledge and experience in healthcare data sharing, cybersecurity, and relevant research methodologies critically evaluated the items to ensure that they effectively measured the key dimensions of data sharing protocols, EHR privacy and security features, and cybersecurity practices.

Reliability, a fundamental aspect of measurement accuracy in research, was meticulously assessed for each tool employed in this study. The data sharing agreement (DSA) framework underwent thorough scrutiny, with the calculation of Cronbach's alpha as a robust indicator of its internal consistency. The results revealed an impressive Cronbach's alpha value of 0.87, signifying a high level of Reliability. Similarly, the electronic health record (EHR) privacy and security assessment tool comprehensively evaluated its internal consistency using Cronbach's alpha. The findings were notably robust, with a calculated alpha value of 0.88. This high degree of internal consistency underscores the tool's Reliability in assessing EHR privacy and security features, indicating that it consistently measures these aspects stably and dependably. The Cronbach's alpha value of 0.88 signifies a strong level of agreement among the tool's items, further enhancing the credibility of the data generated from this instrument.

### Ethical approval and consideration

This study adhered to stringent ethical standards and received approval from the Research Ethics Committee (REC) at the Faculty of Nursing, Zagazig University, Egypt under the code ID/Zu.Nur.REC#:0067﻿.﻿ Nurses were described the study's objectives, methodologies, potential risks, and benefits and provided written, informed consent before participation, signifying their understanding of the study's purpose and their voluntary decision to contribute. Strict confidentiality measures were implemented, ensuring all collected data was anonymised and securely stored to protect participant privacy.

### Statistical analysis

Statistical analysis was conducted using SPSS 26. Descriptive statistics, including counts, percentages, mean scores and standard deviations (mean ± SD), were systematically employed to offer a detailed overview of demographic characteristics and the usage status of the Privacy Impact Assessment (PIA) Tool, Data Sharing Agreement (DSA) Framework, Electronic Health Record (EHR) Privacy and Security Assessment Tool, and National Institute of Standards and Technology (NIST) Cybersecurity Framework. These statistical measures provided a nuanced understanding of the respondents' backgrounds, contributing valuable insights into the diverse composition of the sample and the distribution of tools' utilisation among participants. Spearman's rank correlation coefficient (r) was utilised to unveil significant associations among the tools, highlighting the interconnected nature of these critical constructs within the nursing context.

Furthermore, the study integrated inferential statistics, including ANOVA and t-tests, to add depth to the analysis of the tools. These statistical methods uncovered associations and significant differences related to demographic variables, contributing to a holistic understanding of the factors influencing nurses' attitudes and behaviours towards privacy, security, and data-sharing practices. This multifaceted statistical approach, executed with the aid of SPSS 26, captured the distribution of key attributes and explored relationships and patterns across variables pertinent to the tools' implementation and impact.

## Results

Table 1 shows the demographic profile of study participants. Most participants were between 31 and 40 (45%), followed by those aged 20–30 (35%). Female participants comprised 75% of the sample, while males comprised 25%. Most participants were married (55%), with 40% being single and 5% divorced or in other categories. Regarding job titles, 60% were staff nurses, 20% were nurse managers, 10% were nurse educators, and 10% were nurse practitioners. Experience-wise, 30% had 0–5 years, 25% had 6–10 years, 20% had 11–15 years, and 25% had over 16 years of nursing experience. Regarding qualifications, 40% held a diploma, 35% a bachelor's degree, 20% a master's degree, and 5% a doctorate. A significant majority had received training on confidentiality (70%), while half had training on digital health technologies. The primary language of communication was Arabic (80%), with English used by 20% of the participants.

**Table 1:** Demographic profile of study participants

Variable	Number of Participants	Frequency (%)
**Age**		
20–30 years	52	35%
31–40 years	68	45%
41–50 years	23	15%
51 + years	7	5%
**Gender**		
Male	38	25%
Female	112	75%
**Marital Status**		
Single	60	40%
Married	83	55%
Divorced/Other	7	5%
**Current Job Title/Position**		
Staff Nurse	90	60%
Nurse Manager	30	20%
Nurse Educator	15	10%
Nurse Practitioner	15	10%
**Years of Nursing Experience**		
0–5 years	45	30%
6–10 years	38	25%
11–15 years	30	20%
16 + years	37	25%
**Highest Nursing Qualification**		
Diploma	60	40%
Bachelor's Degree	52	35%
Master's Degree	30	20%
Doctorate	8	5%
**Training on Confidentiality**		
Yes	105	70%
No	45	30%
**Training on Digital Health Tech**		
Yes	75	50%
No	75	50%
**Primary Language of Communication**		
Arabic	120	80%
English	30	20%

Table 2 presents the mean scores and standard deviations for the components of the HIPAA Compliance Checklist. The results indicate that the highest compliance was observed in Administrative Safeguards, with a mean score of 3.8 ± 0.5. Technical Safeguards follow this with a mean score of 3.7 ± 0.6. Physical Safeguards had a mean score of 3.5 ± 0.7. In contrast, Organisational Requirements had a mean score of 3.6 ± 0.8. The overall total score was 85 with a standard deviation of 10, suggesting generally high compliance with some variability among the components.

**Table 2:** Health insurance portability and accountability act (HIPAA) compliance checklist

Component	Mean Score	Standard Deviation
Administrative Safeguards	3.8	0.5
Physical Safeguards	3.5	0.7
Technical Safeguards	3.7	0.6
Organisational Requirements	3.6	0.8
**Total Score**	**85**	**10**

Table 3 displays the mean scores and standard deviations for each Privacy Impact Assessment (PIA) Tool section. Across all sections, high scores were observed, indicating robust compliance with privacy standards. Project Descriptions received the highest mean score of 4.5 ± 0.3, reflecting clear and comprehensive project documentation. Data Storage and Security Strategies also scored a mean of 4.4 ± 0.4, highlighting strong measures for protecting data integrity and security. The total score of 30 ± 3 underscores overall high adherence to privacy protocols, albeit with some variability across specific assessment criteria.

**Table 3:** Privacy impact assessment (PIA) tool

Section	Mean Score	Standard Deviation
Project Descriptions	4.5	0.3
Data Collection Methods	4.3	0.4
Data Usage and Sharing Practices	4.2	0.5
Data Storage and Security Strategies	4.4	0.4
Identification of Privacy Risks	4.3	0.5
Risk Mitigation Methods	4.3	0.4
**Total Score**	**30**	**3**

Table 4 presents the mean scores and standard deviations (mean ± SD) for each Data Sharing Agreement (DSA) Framework component. The assessment reveals solid compliance across all components, with Data Protection Measures achieving the highest mean score of 4.0 and a standard deviation of 0.4, indicating robust safeguards for data security. Purpose of Data Sharing and Roles and Responsibilities both received a mean score of 3.8, demonstrating clarity in defining the objectives and delineating roles in data-sharing activities. Consent Management and Data Use and Retention Terms also scored well, reflecting comprehensive practices in managing consent and outlining data use and retention terms. The total score of 20 with a standard deviation of 2 indicates strong adherence to data-sharing protocols, with minor variability in assessment outcomes.

**Table 4:** Data sharing agreement (DSA) framework

Component	Mean Score	Standard Deviation
Purpose of Data Sharing	3.8	0.4
Types of Data to be Shared	3.7	0.5
Roles and Responsibilities	3.8	0.3
Data Protection Measures	4.0	0.4
Consent Management	3.9	0.4
Data Use and Retention Terms	3.8	0.3
**Total Score**	**20**	**2**

Table 5 presents the mean scores and standard deviations for each Electronic Health Record (EHR) Privacy and Security Assessment Tool component. Access Controls received the highest mean score of 4.4, indicating strong implementation of measures to control access to patient information. Encryption Methods and Data Integrity Measures scored 4.3, highlighting robust practices in securing and maintaining the integrity of EHR data. Audit Trail Functionalities and Incident Response Protocols scored 4.2, indicating effective mechanisms for tracking access to records and responding to security incidents. The total score of 22 ± 1.5 suggests high overall compliance with EHR privacy and security requirements, with minimal variability in assessment outcomes.

**Table 5:** Electronic health record (EHR) privacy and security assessment tool

Component	Mean Score	Standard Deviation
Access Controls	4.4	0.3
Encryption Methods	4.3	0.4
Audit Trail Functionalities	4.2	0.4
Data Integrity Measures	4.3	0.3
Incident Response Protocols	4.2	0.3
**Total Score**	**22**	**1.5**

Table 6 displays the mean scores and standard deviations for each function of the National Institute of Standards and Technology (NIST) Cybersecurity Framework. The framework is designed to enhance cybersecurity practices across healthcare settings, focusing on five core functions: Identify, Protect, Detect, Respond, and Recover. Protect achieved the highest mean score of 3.8, indicating strong implementation of measures to protect against cybersecurity threats. Identify, Respond, and Recover scored similarly at 3.7, highlighting robust capabilities in identifying assets, responding to incidents, and recovering from cybersecurity events. Detect scored slightly lower at 3.6, suggesting areas for potential improvement in detecting and mitigating threats. The total score of 18 ± 2 reflects generally effective cybersecurity practices with moderate variability in implementation across functions.

**Table 6:** National institute of standards and technology (NIST) cybersecurity framework

Function	Mean	Standard Deviation
Identify	3.7	0.4
Protect	3.8	0.3
Detect	3.6	0.5
Respond	3.7	0.4
Recover	3.7	0.4
**Total Score**	**18**	**2**

The correlation matrix (Table 7) reveals significant relationships among key frameworks for assessing healthcare data security and privacy measures. These tools include the Health Insurance Portability and Accountability Act (HIPAA) Compliance Checklist, Privacy Impact Assessment (PIA) Tool, Data Sharing Agreement (DSA) Framework, Electronic Health Record (EHR) Privacy and Security Assessment Tool, and the National Institute of Standards and Technology (NIST) Cybersecurity Framework. Strong positive correlations were found between HIPAA Compliance and both EHR Privacy and Security (*r* = 0.70, *p* < 0.05) and NIST Cybersecurity Framework (*r* = 0.55, *p* < 0.05), indicating that adherence to HIPAA regulations often coincides with robust electronic health record protections and cybersecurity practices. The PIA Tool demonstrated moderate positive correlations with the DSA Framework (*r* = 0.55, *p* < 0.05) and EHR Privacy and Security (*r* = 0.60, *p* < 0.05), underscoring the alignment between thorough privacy impact assessments and effective data sharing agreements and EHR security measures. These findings highlight the interconnectedness of regulatory compliance and proactive privacy measures in ensuring comprehensive healthcare data protection across organisational settings.

**Table 7:** Correlation matrix of HIPAA compliance checklist, privacy impact assessment (PIA) tool, data sharing agreement (DSA) framework, electronic health record (EHR) privacy and security assessment tool, and national institute of standards and technology (NIST) cybersecurity framework

Tool	HIPAA Compliance	PIA	DSA	EHR Privacy	NIST Cybersecurity
HIPAA Compliance	1.00				
PIA	0.65^∗^	1.00	0.55^∗^	0.60^∗^	0.50^∗^
DSA Framework	0.60^∗^	0.55^∗^	1.00	0.45^∗^	0.40^∗^
EHR Privacy and Security	0.70^∗^	0.60^∗^	0.45^∗^	1.00	0.75^∗^
NIST Cybersecurity Framework	0.55^∗^	0.50^∗^	0.40^∗^	0.75^∗^	1.00

## Discussion

Nurses are pivotal in the healthcare system, and their expertise spans various domains, from clinical practice to administrative roles, influencing the quality and delivery of healthcare services. In recent years, the evolving healthcare landscape has underscored the need for nurses to navigate complex challenges such as patient privacy, data security, and regulatory compliance, are crucial for safeguarding patient information and maintaining trust and integrity within healthcare settings. This study explored the efficacy of several frameworks and tools designed to enhance data privacy and security measures, aiming to empower nurses with comprehensive strategies that align with regulatory standards and promote optimal patient care outcomes [46, 47].

The high mean scores in administrative safeguards (mean = 3.8, SD = 0.5) and technical safeguards (mean = 3.7, SD = 0.6) reflected a strong commitment to data privacy and security within the healthcare sector. These findings indicated that some healthcare organisations are implementing measures to secure electronic protected health information (ePHI) and manage access controls effectively. However, there remains variability that needs addressing. The lower scores in physical safeguards (mean = 3.5, SD = 0.7) and organisational requirements (mean = 3.6, SD = 0.8) highlight areas where further attention is needed. The variability in these scores suggests potential challenges in implementing physical security measures and ensuring consistent policy documentation and workforce training. Previous studies highlighted the importance of comprehensive physical security measures and consistent organisational policies in maintaining overall compliance [48–56].

Regarding the Privacy impact assessment, high scores in project descriptions (mean = 4.5, SD = 0.3) and data storage and security strategies (mean = 4.4, SD = 0.4) suggested thorough documentation and robust security measures are in place, effectively identifying and mitigating privacy risks associated with new projects and data handling practices. However, the variability in scores across different sections of the PIA Tool indicated a need for continuous improvement in data usage, sharing practices, and risk mitigation methods, where consistent implementation may vary. These findings are consistent with previous studies that emphasised the need for comprehensive project documentation and secure data handling practices [57–65].

Concerning the data sharing agreement, the high scores in Data Protection Measures (mean = 4.0, SD = 0.4) indicated robust safeguards for data security. The purpose of data sharing and roles and responsibilities also performed well, reflecting clear definitions of data sharing objectives and roles. However, the moderate score variability indicated challenges in uniformly implementing consent management practices and data use terms. Prior studies also support the critical role of well-defined data-sharing agreements in balancing data utility and privacy protection [66–69].

In terms of electronic health record privacy and security assessment, high scores in access controls (mean = 4.4, SD = 0.3), encryption methods, and data integrity measures (mean = 4.3) reflected advancements in technology and policies aimed at enhancing data protection in healthcare settings, highlighting effective implementation of access management protocols. However, the minor variability in scores suggested room for improvement in incident response protocols. These findings were consistent with literature advocating for robust access controls and encryption methods to mitigate risks associated with EHR breaches [70–75].

With respect to the NIST Cybersecurity Framework**,** the high scores in the Protect function (mean = 3.8) indicated strong measures to protect healthcare information systems from cybersecurity threats. The identify, respond, and recover functions also scored well (mean = 3.7), highlighting robust capabilities in identifying assets, responding to incidents, and recovering from cyber-attacks. However, the slightly lower score in the detect function (mean = 3.6) suggested areas for improvement in detecting and mitigating cybersecurity threats. These results were supported by research emphasising the effectiveness of the NIST framework in enhancing cybersecurity resilience across various sectors, including healthcare [76–82].

Moreover, the current study revealed significant relationships among key frameworks used to assess healthcare data security and privacy measures, underscoring the interconnectedness of regulatory compliance efforts and proactive privacy measures. For instance, strong positive correlations were found between HIPAA Compliance and both EHR Privacy and Security (*r* = 0.70, *p* < 0.05) and the NIST Cybersecurity Framework (*r* = 0.55, *p* < 0.05), indicating that adherence to HIPAA regulations often coincides with robust electronic health record protections and cybersecurity practices. The PIA Tool demonstrated moderate positive correlations with the DSA Framework (*r *= 0.55, *p* < 0.05) and EHR Privacy and Security (*r *= 0.60, *p* < 0.05), highlighting the alignment between thorough privacy impact assessments and effective data sharing agreements and EHR security measures. These findings suggested that while certain frameworks complement each other well, there may be specific areas where improvements could enhance overall data security posture [83–85].

### Study Limitations

This study has several notable limitations. Firstly, the cross-sectional design captures data at a single point in time, which may not fully reflect the dynamic nature of digital healthcare environments and evolving privacy challenges. Future research could address this by employing a longitudinal design to track how privacy and care coordination evolve with changes in technology and regulations. Secondly, the study was conducted at a single hospital, which may limit the generalizability of the findings to other healthcare settings with different digital infrastructures and privacy practices. Including multiple healthcare settings with diverse digital systems and privacy practices in future studies could enhance the applicability of the findings.

Additionally, the reliance on self-reported data from nurses introduces potential response bias, as participants may overstate their adherence to privacy and security protocols, resulting in inflated compliance rates. The exclusion of nurses on extended leave or those in administrative roles also limits the study's comprehensiveness. These groups might have unique insights or experiences related to confidentiality and care coordination that are not captured in the current study. Finally, while the study used validated tools, the rapid evolution of digital health technologies means that these tools may quickly become outdated. The changing landscape of technology and privacy standards can affect the relevance and accuracy of the assessment instruments. Addressing these limitations in future studies will provide more comprehensive understanding of privacy and care coordination in digital healthcare environments and improve the relevance and applicability of the findings across different contexts and periods.

## Conclusion and recommendations

In conclusion, this study underscores the crucial role of nurses in ensuring robust data privacy and security within healthcare settings. The findings reveal high compliance with HIPAA regulations, particularly in administrative and technical safeguards, and strong performance in project descriptions and data storage strategies. The adherence to data privacy and sharing protocols, effective EHR security measures, and alignment with the NIST Cybersecurity Framework reflect a comprehensive approach to data protection. However, the variability in certain areas, such as physical safeguards, organisational requirements, and detection measures, highlights the need to continuously enhance data security practices to maintain the integrity and trust essential in healthcare. Investing in continuous training programs for nurses is crucial. Healthcare organisations should provide regular, specialised training addressing emerging privacy regulations, cybersecurity threats, and best practices. Upgrading physical security measures, such as access controls and surveillance, and ensuring that all organisational policies and procedures are up-to-date with current regulations will help achieve comprehensive HIPAA compliance.

Another key recommendation is to standardise and enhance consent management practices and data use terms. Organisations should develop clear, standardised consent forms and data use agreements, implement automated systems for tracking and managing consent, and regularly review and update these policies to reflect regulation changes. Additionally, conducting regular audits and updating detection measures is vital for improving overall cybersecurity posture. Lastly, fostering a culture of continuous improvement and proactive privacy management within healthcare organisations is essential. Encouraging open communication about privacy and security concerns, rewarding compliance and proactive measures, and engaging staff in regular discussions about privacy and security initiatives will contribute to a robust privacy management culture.

### Study Implications

The findings of this study offer several actionable insights for healthcare practice, policy, and future research.

- Healthcare Practice: The study highlights the critical need for continuous and comprehensive training for nurses on digital health privacy and security protocols. Specific recommendations include developing targeted training programs that address emerging privacy threats and technologies. Additionally, integrating privacy and security training into onboarding processes for new staff can ensure that all personnel are up-to-date with best practices from the start.
- Policy: Policymakers should prioritise the development of detailed guidelines that address the specific challenges posed by these technologies, such as data sharing and electronic health records. Recommendations include establishing clear standards for data encryption, access controls, and consent management. Regular policy reviews and updates are necessary to keep pace with technological advancements and ensure ongoing protection of patient confidentiality.
- Future Research: Longitudinal studies are needed to assess how implementing digital health technologies impacts patient privacy and care coordination over time. Future studies could also focus on developing and validating new assessment tools that reflect the latest technological advancements and privacy challenges. Investigating the role of interdisciplinary approaches, combining insights from cybersecurity experts and healthcare practitioners, could further enhance privacy and security measures in digital health environments.

## Data Availability

Data sharing is not applicable to this article as no datasets were generated or analysed during the current study.

## Data availability

No datasets were generated or analysed during the current study.
